# No evidence for niche competition in the extinction of the South American saber-tooth species

**DOI:** 10.1038/s44185-024-00045-7

**Published:** 2024-06-05

**Authors:** Roniel Freitas-Oliveira, Matheus S. Lima-Ribeiro, Levi Carina Terribile

**Affiliations:** 1 Programa de Pós-Graduação em Ecologia e Evolução, Instituto de Ciências Biológicas, Universidade Federal de Goiás, Goiânia, GO Brazil; 2 Laboratório de Macroecologia, Universidade Federal de Jataí, UFJ, Jataí, GO Brazil; 3 National Institute for Science and Technology (INCT) in Ecology, Evolution and Biodiversity Conservation, Goiânia, Goiás Brazil

**Keywords:** Climate-change ecology, Community ecology, Macroecology, Palaeoecology

## Abstract

The end of South American isolation during the Great American Biotic Interchange (GABI) promoted the contact between South and North American saber-tooth forms that evolved in isolation. This contact may have driven saber-tooth species to a competitive interaction, resulting in the extinction of the South American saber-tooth form. Here, we used paleoclimatic data to compare the climatic niche of the saber-tooth forms *Thylacosmilus atrox* (from South America), *Smilodon fatalis*, and *Smilodon populator* (both originally from North America). We evaluated niche width, overlap, and similarity to infer potential geographic distribution overlap and competition between these North and South American predators. To do so, we obtained the climatic variables from sites where occurrence fossil records were available. Our results suggest that *T. atrox* had a narrower climatic niche compared to *Smilodon* species. Although we found a significant climatic niche overlap and similarity between *S. fatalis* and *T. atrox*, it seems unlikely that both species have co-occurred. Low niche overlap and similarity between *T. atrox* and *S. populator* dismiss competitive interaction between them. Moreover, climatic niche and low tolerance for environmental changes may have been the cause of the South American saber-tooth extinction.

## Introduction

The South American continent remained in isolation for a long time over the Cenozoic, resulting in a peculiar mammal assemblage structure, such as the endemic metatherian apex predators^1,2^. However, the Great American Biotic Interchange (GABI) ended the South American isolation and promoted the arrival of the North American carnivores^3^. The GABI changed expressively the history of mammals in South America, where almost half of its current fauna has North American origin^4^. Even so, it is still unknown how this fauna interacted when they came into connection.

The contact between faunas that evolved in isolation was already pointed out to be responsible for South American mammalian extinctions^5^. The order Carnivora (Eutheria) arrived in South America between 9 and 3 Ma (pre-GABI, see^6^), the time at which the extinction rate of Sparassodonta (Methateria) increased^7^. Since carnivores and sparassodonts are typical examples of evolutionary convergence (e.g., both have fox-like and saber-toothed forms^2,8^), it has been proposed that Sparassodonta became extinct during the Plio-Pleistocene due to competition with placental Carnivora from North America^4,9^, a classic example of the competitive exclusion principle, by which species with identical niches cannot coexist indefinitely^10^. This principle is based on the fact that resources are limited to some extent, and species that exploit similar resources naturally compete for them^11^. Under such a scenario, the weaker competitor perishes, and the more skilled one survives.

Recent studies have pointed out that, although placental carnivores replaced the Sparassodonta in South America, there is no clear evidence of competition between them^7,12,13^. We could infer that competition was the cause of extinction if the species have indeed co-occurred in time and space, and played a similar ecological function, which seems to be the case for South American carnivores and sparassodonts. Patterns of species co-occurrence in space and time can be investigated from the perspective of climatic niche overlap or differentiation, however, this has not been done hitherto. On the other hand, the extinction of Sparassodonta may have been caused by other events, such as the extinction of their prey and environmental changes related to the Andean uplift, atmospheric CO², temperature, and sea level^7,13^.

Here, we used paleoclimatic data to compare the climatic niche of two saber-toothed groups, Sparassodonta, represented by the species *Thylacosmilus atrox* (Riggs, 1933), and Carnivora, represented by two species of *Smilodon* (Lund, 1842): *S. fatalis* (Leidy, 1868) and *S. populator* (Lund, 1842). These two groups of large-sized predators inhabited open vegetation areas of South America ~6 Ma^4^ and preyed upon large herbivores^2,12,14–16^. We evaluated both groups’ niche width, overlap, and similarity. If there is climatic niche overlap between *T. atrox* and *Smilodon*, these groups could have co-occurred, and the competitive exclusion of Sparassodonta cannot be discarded. We also expect higher climatic niche overlap between *T. atrox* and *S. populator* because *S. populator* was more widely distributed in South America in comparison with *S. fatalis*^17^. On the other hand, the absence of niche overlap and niche similarity makes way for other causes (such as environmental changes) for Sparassodonta extinction.

## Results

### Climatic niche width, similarity, and overlap

**Fig. 1:**
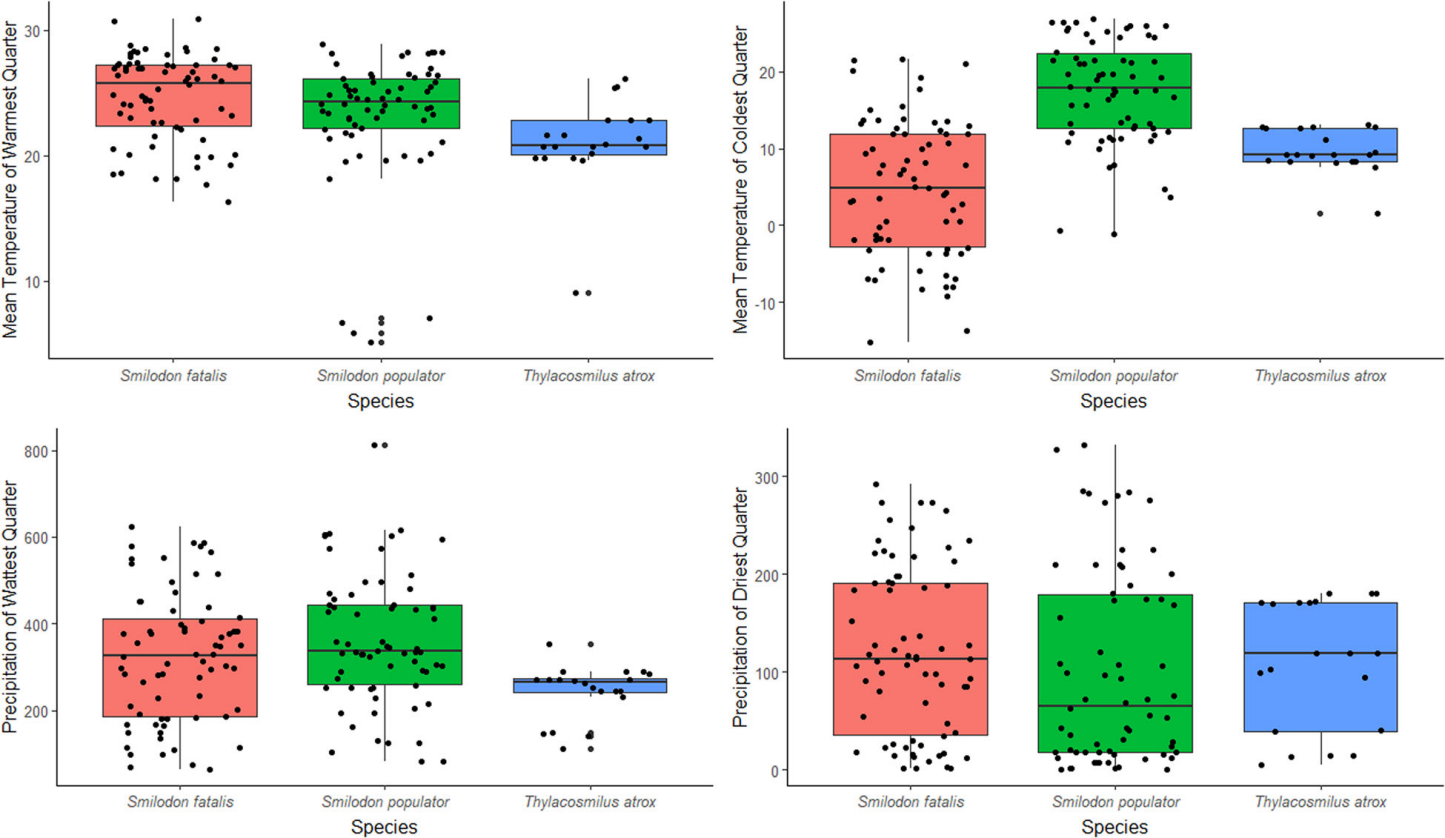
Climatic variation from the occurrence fossil records of South and North American saber-tooth predators.

*Thylacosmilus atrox* presented a narrower climatic niche width in comparison to *S. fatalis* and *S. populator*, except for the mean temperature of the warmest quarter, which is wider in *T. atrox*. *Smilodon fatalis* presented a narrower climatic niche in comparison to *S. populator*, except for the mean temperature of coldest quarter (Fig. 1). Proportionally, the climatic niche width of *T. atrox* vary from 0.31–0.6 in comparison to *S. fatalis* and *S. populator*, except for mean temperature of warmest quarter, which was wider in *T. atrox* than in *S. fatalis* and corresponds to 0.71 – *S. populator*’s niche width (Table 1).

**Fig. 2:**
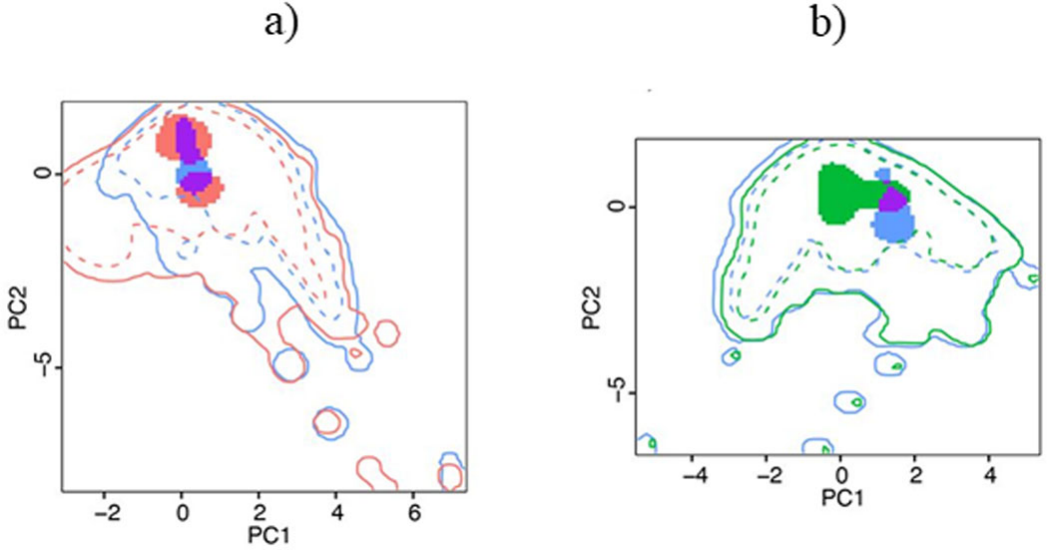
Climatic niche overlap for saber-tooth predators. Niche overlap between *T. atrox* (in blue) and *S. fatalis* (in red) (**a**), and between *T. atrox* and *S. populator* (in green) (**b**). Purple color indicates overlapping between two species. The solid and dashed lines represent 100% and 50% of the available climatic space, respectively.

The results of niche overlap and similarity were quite similar in both time intervals (5 Ma and between 3.8 and 1.8 Ma). Thus, hereafter we only kept the results for the time interval between 3.8 and 1.8 Ma (the results for the whole period of 5 Ma are provided in Supplementary information). The similarity of the climatic niche between *T. atrox* and *S. fatalis* was higher than expected from random (*p* = 0.005 for *Schoener’s D*, and *p* < 0.005 for *Hellinger’s I*), but the similarity between *T. atrox* and *S. populator* did not differ from random expectations (*p* = 0.14 for *Schoener’s D*, and *p* = 0.15 for *Hellinger’s I*). Additionally, there was considerable niche overlap between *T. atrox* and *S. fatalis* (*Schoener’s D* = 0.34 and *Hellinger’s I* = 0.52 Fig. 2a), and low niche overlap between *T. atrox* and *S. populator* (*Schoener’s D* = 0.1 and *Hellinger’s I* = 0.14, Fig. 2b).

**Table 1:** Niche width proportion of *Thylacosmilus atrox* concerning *Smilodon fatalis* and *Smilodon populator*

Bioclimatic	*Smilodon fatalis*	*Smilodon populator*
Mean temperature of warmest quarter	1.16	0.71
Mean temperature of coldest quarter	0.31	0.41
Mean precipitation of wettest quarter	0.43	0.33
Mean precipitation of driest quarter	0.60	0.52

Values higher than 1 indicate that the climatic niche of *T. atrox* was wider.

## Discussion

**Fig. 3:**
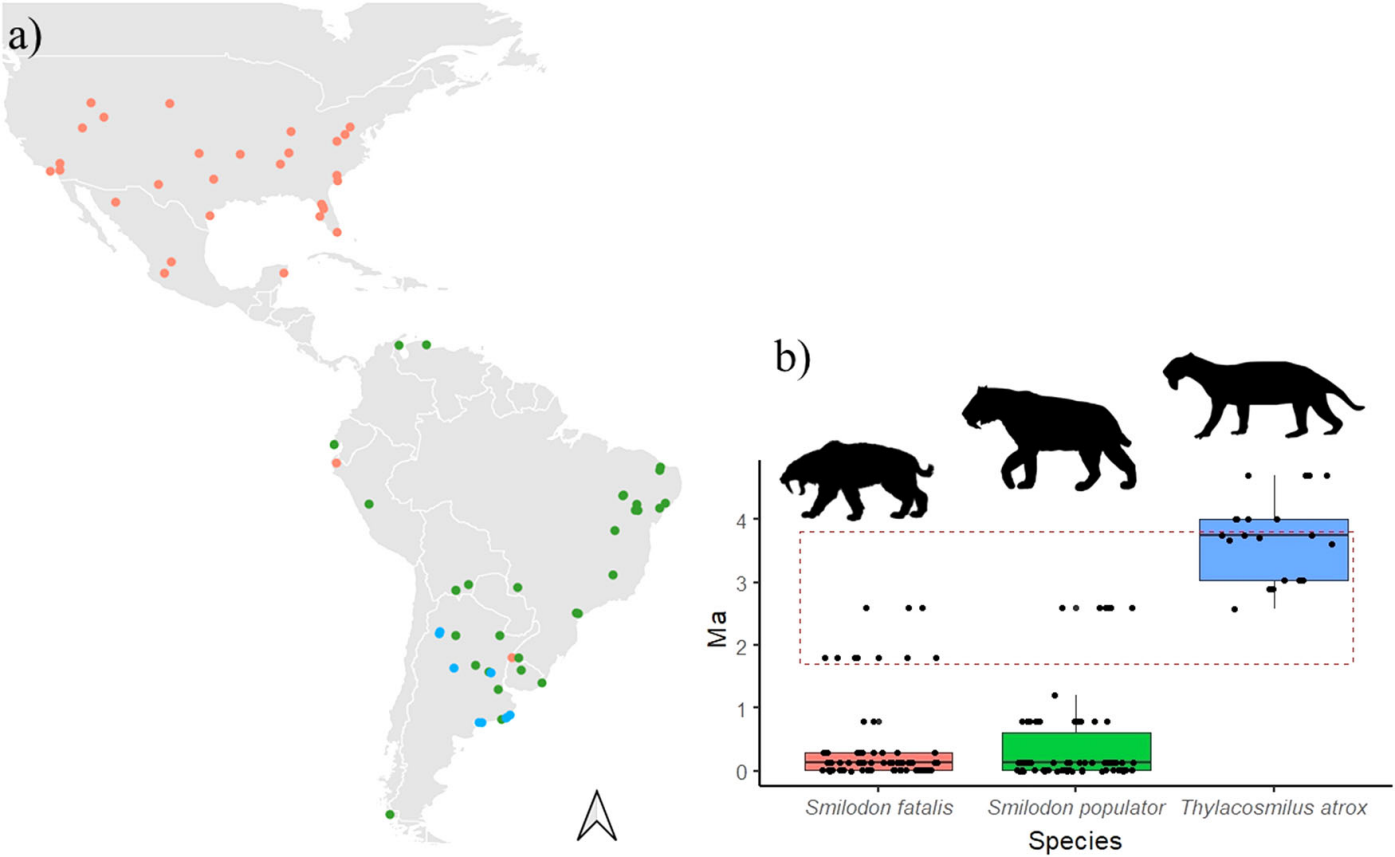
Occurrence records of Saber-tooth predators. **a** Spatial and **b** temporal (in millions of years—Ma) distribution of the occurrence records of *Thylacosmilus atrox, Smilodon fatalis, and Smilodon populator* in the American continent. The red dashed box indicates the time slice of 2 Ma (from 3.8 to 1.8 Ma) for which niche overlap and similarity were estimated. The silhouettes were obtained from PhyloPic https://www.phylopic.org/. *Thylacosmilus atrox* was designed by Zamices (Julián Bayona), under the Creative Commons Attribution-NonComercial 3.0 Unported license https://creativecommons.org/licenses/by-nc/3.0/ no changes were made. *Smilodon fatalis* was designed by Steven Traver, under the Creative Commons CC0 1.0 Universal Public Domain Dedication license (https://creativecommons.org/publicdomain/zero/1.0/) no changes were made. *Smilodon* (considered here as *S. populator* for illustration purposes) was designed by Margot Michaud under Creative Commons CC0 1.0 Universal Public Domain Dedication license (https://creativecommons.org/publicdomain/zero/1.0/) no changes were made.

We found no evidence of competition between *T. atrox* and *S. populator*, suggesting they occurred in different climatic conditions and, probably, did not overlap geographically. On the other hand, although the niche similarity and overlap between *T. atrox* and *S. fatalis* could indicate a competition signal, these species probably also did not co-occur (the distribution of *T. atrox* was restricted to Argentina, where no record for *S. fatalis* is known, Fig. 3a^17,18^). Rather, the niche similarity between them could be due to the broader climatic niche of *S. fatalis*, which could comprise climatic conditions also suitable for *T. atrox*, although in distinct geographic areas.

We found that the South American *T. atrox* had a narrower climatic niche compared to the North American *Smilodon* species. However, the interpretation of niche width for *T. atrox* should be cautious due to the lack of information about the distribution of this species (*n* = 20 occurrence records), which may have contributed to the narrower niche estimate found here. Despite this, our results corroborate previous works^16^, which found a narrower niche diet for *T*. *atrox* in comparison with *Smilodon*. Species with narrower climatic niche width may have lower tolerance to climate change, and narrower temperature niche width has been associated with species extinction^19^. Also, narrower thermal tolerance has already been related to climatic change vulnerability in mountain species with low dispersion capacity, and in species with long generation times^20,21^ (e.g., large-sized mammals). Therefore, the more restrictive distribution of *T. atrox* in the southern portion of South America (Fig. 3), and its narrower climatic niche together with its larger body size (117.36 kg^2,15^) suggest that this species could have been more vulnerable to environmental changes than its potential competitors *Smilodon* species.

Our results corroborate previous results that failed to find competition evidence between Sparassodonta and Carnivora^2,7,12,13,22^. Thus, other factors still unknown may have been responsible for the South American apex predator extinction. Indeed, recent studies agreed that high extinction rates in Sparassodonta started in the Miocene (16 Ma^13^, 17 Ma^7^) and remained until the group extinction in Pliocene^13^. Changes in regional landscape due to Andean uplift, atmospheric CO_²,_ and sea level were pointed as the cause for Sparassodonta extinction^7,13^. Here, we provide additional support for these causes, showing that *T. atrox* had a narrower climatic niche than the other species investigated herein and, therefore, could have been more vulnerable to climatic change^20,21^.

It should be highlighted that several caveats may affect the interpretation of our results. We are dealing with fossil information for the last 5 Ma, with inaccurate dates and clear distribution gaps throughout the study area (Fig. 3a). Furthermore, the narrowed time slice from 3.8 to 1.8 Ma reduced the number of occurrence records available for niche overlap analyses, precluding more thinned analyses (e.g., glaciation and interglaciation fluctuations). Also, the coarse resolution (1 degree of resolution^20^) of climate data available for that period has limitations for investigating species interaction locally. Despite this, the analysis including all available fossil records showed similar results, thus providing additional support to the patterns unveiled in this study. Indeed, our results add up with previous findings of no competitive interaction between sparassodonts and carnivores, thus increasing the body of evidence for the potential causes of South American saber-tooth extinction.

## Methods

### Occurrence and paleoclimatic data

We obtained fossil occurrence data of *T. atrox*, *S. fatalis*, and *S. populator* spanning from 5 Ma to the present, the period for which the climate data was available (see below), from The Paleobiology Database (PBDB https://paleobiodb.org). Occurrence records of *T. atrox* were also obtained from literature^7^ due to the low number of occurrences available in the PBDB. We retrieved 156 occurrence records (20 for *T. atrox*^7,23^, 70 for *S. fatalis* and 66 for *S. populator*^24^, Fig. 3). We extracted climate values for each fossil occurrence for the only four bioclimatic variables available in the PALEO-PGEM-Series^25^ (1-degree of resolution): mean temperature of warmest quarter, mean temperature of coldest quarter, mean precipitation of wettest quarter, and mean precipitation of driest quarter. We used the “raster” package^26^ for extracting climate data.

### Climatic niche width, similarity, and overlap

To compare the climatic niche of *T. atrox* and *Smilodon* species, we calculated the niche width, i.e., the difference between the highest and the lowest values for each bioclimatic variable for each species. Then, we calculated the proportion of the *T. atrox* niche width concerning the *Smilodon* species, dividing the width value of *T. atrox* by the width value of both *Smilodon* species.

The temporal resolution of 5 Ma can be considered too coarse to interpret potential changes in species niche due to the magnitude of climate variability during the Plio-Pleistocene. Thus, for niche overlap and similarity analysis, we narrowed the time slice to the period between 3.8 and 1.8 Ma (Fig. 3b, red dashed box), which encompasses a period of higher overlap among the records for all species analyzed here. Within this period of 2 Ma we keep 12 fossil records for *T. atrox*, 10 for *S. fatalis*, and 6 for *S. populator* (Fig. 3b). We also estimated the niche metrics based the whole period of 5 Ma to assess the consistency of the results through different time intervals (i.e., 2 Ma and 5 Ma). In this case, we applied a bootstrap approach to account for the differences in the number of fossil records among the species (from 20 for *T. atrox* to 70 for *S. fatalis*; see Supplementary information).

We conducted the niche overlap analyses using “ecospat” package^27–30^, on R environment^31^. The distribution of *T. atrox* and *S. populator* fossil records was restricted to South America, while *S. fatalis* had fossil records throughout the whole American continent (Fig. 3). For both regions (South America and the whole American continent) we created two environmental grids, one composed by the fossil records and the other by the background area. Therefore, the background area for *T. atrox* and *S. populator* was South America, while for *S. fatalis* the background area was the American continent. We obtained background areas for each year of the occurrence records for each species.

We performed a Principal Component Analysis (PCA) using the “dudi.pca” function of the ade4 package^32^ using the environmental grids, one PCA for each background area. Subsequently, we used the two first PCA axes to create a kernel density estimation using the “ecospat.grid.clim.dyn” function. We then tested the similarity between the niches using the “ecospat.niche.similarity.test” function, from which we extracted the probability of the similarity being higher than randomly, for *Schoener’s D* and *Hellinger’s I indices*. We also calculated the *Schoener’s D* and *Hellinger’s I indices* to assess species’ niche overlap. Finally, we plot the niches in environmental space using “ecospat.plot.niche.dyn”. These analyses were performed separately for each species interaction: between *T. atrox* and *S. fatalis*, and between *T. atrox* and *S. populator*.

### Supplementary information

Supplementary Information

## Data Availability

No datasets were generated or analysed during the current study.
