# Supplementary material for: No evidence for niche competition in the extinction of the South American saber-tooth species
Source: NPJ Biodivers. 2024 Jun 5;3:11. doi: 10.1038/s44185-024-00045-7 (PMC11332042; doi:10.1038/s44185-024-00045-7)
Supplement: Supplementary file 1 — Supplementary Information [file 44185_2024_45_MOESM1_ESM.pdf]

No evidence for niche competition in the extinction of the South American saber-tooth species

Roniel Freitas-Oliveira[1,2], Matheus S. Lima-Ribeiro[3], Levi Carina Terribile[2]

[1]Programa de Pós-Graduação em Ecologia e Evolução, Instituto de Ciências Biológicas, Universidade Federal de Goiás, Goiânia, GO, Brazil

[2]Laboratório de Macroecologia, Universidade Federal de Jataí, UFJ, Jataí, GO, Brazil

[3]National Institute for Science and Technology (INCT) in Ecology, Evolution and Biodiversity Conservation, Goiânia, Goiás, Brazil.

[*]Corresponding author: Roniel Freitas-Oliveira, freitasronielbio@gmail.com

Supplementary information

Niche metric estimates for 5 Ma

The niche overlap analysis across the whole 5 Ma period provided similar results as for the 3.8 – 1.8 Ma interval. The climatic niche similarity was significant between *T. atrox* and *S. fatalis* (p=0.001 for *Schoener's D*, and p< 0.003 for *Hellinger's I*), but not between *T. atrox* and *S. populator* (p=0.10 for *Schoener's D*, and p=0.05 for *Hellinger's I*). The niche overlap between *T. atrox* and *S. fatalis* (*Schoener's D= 0.41* and *Hellinger's I= 0.62*, Supplementary Figure 1a) was higher than between *T. atrox* and *S. populator,* (*Schoener's D=0.26* and *Hellinger's I=0.47*, Supplementary Figure 1b).

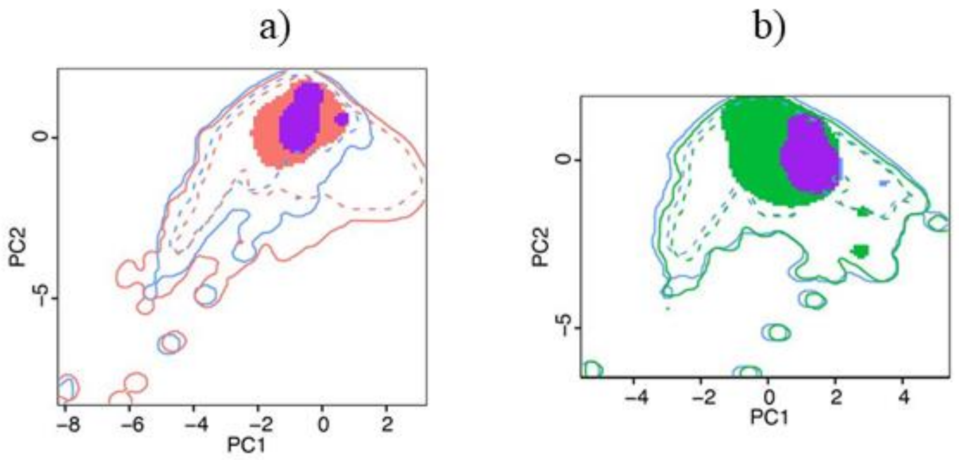

Supplementary Figure 1. Climatic niche overlap for saber-tooth predators. Niche overlap between *T. atrox* (in blue) and *S. fatalis* (in red) (a), and between *T. atrox* and *S. populator* (in green) (b) for the last 5 Ma. Purple color indicates overlapping between two species. The solid and dashed lines represent 100% and 50% of the available climatic space, respectively.

Due to the low number of fossil records for *T. atrox*, which could hamper climatic niche estimation and comparisons with the other species, we used a bootstrap method to randomly resample 20 occurrence records for *Smilodon* species (the same amount of occurrence records available for *T. atrox*) and estimate the niche overlap metrics between *T. atrox* and *Smilodon* species from these subsampled records. We repeated this process 100 times and calculated the probability (p-value) of the estimated niche overlap being different from the randomization estimates. The p-value here is the proportion of the niche overlap values from the bootstrap that were higher than the estimated ones.

The bootstrap analysis indicated that the niche overlap between *T. atrox* and *S. fatalis* did not differ when fossil records for *S. fatalis* were subsampled (*Schoener's D* p=0.48, *Hellinger's I* p< 0.46, Supplementary Figure 4a and b). The same pattern was found for *T. atrox* and *S. populator* (*Schoener's D* p=0.33, *Hellinger's I* p< 0.22, Supplementary Figure 4c and d). This result indicates that the pattern found here was not affected by the differences in the sample size among the species.

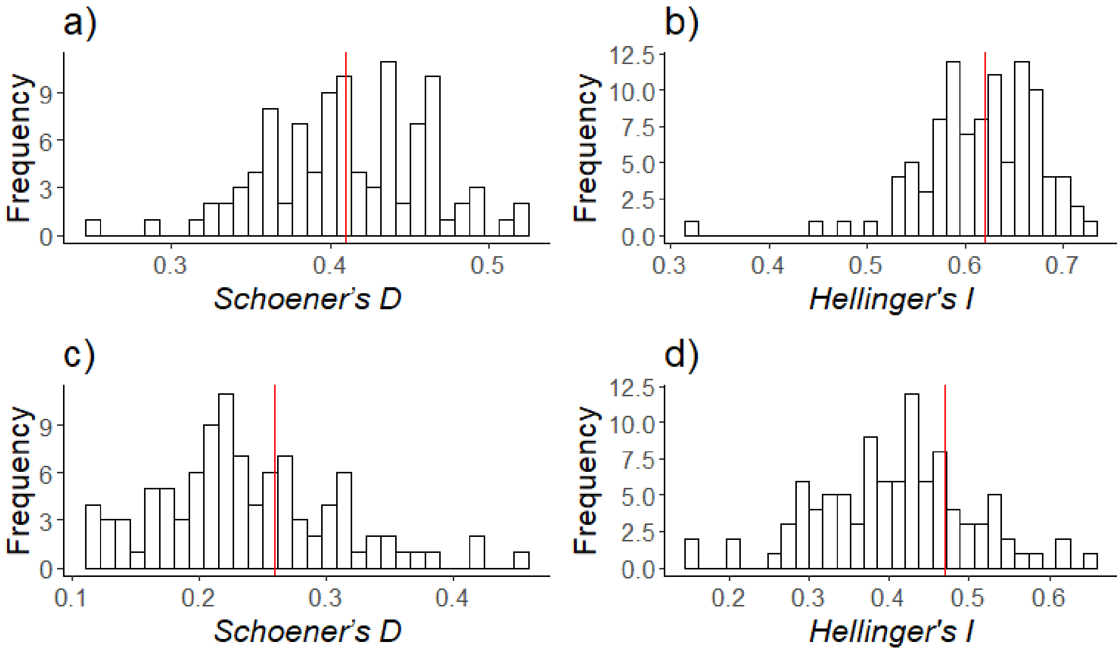

Supplementary Figure 2. Frequency distributions of climatic niche overlap values from bootstrap analyses for the saber-tooth predators.

Frequency distribution for *Thylacosmilus atrox* x *Smilodon fatalis* (a) and (b), and *T. atrox* x *S. populator* (c) and (d). The red line represents the estimated *Schoener's D* and *Hellinger's I values.*
